# Effectiveness of self-administered mirror therapy on upper extremity impairments and function of acute stroke patients: study protocol

**DOI:** 10.1186/s13063-021-05380-9

**Published:** 2021-07-09

**Authors:** Venkadesan Rajendran, Deepa Jeevanantham, Céline Larivière, Ravinder-Jeet Singh, Lisa Zeman, Padma Papuri

**Affiliations:** 1 Health Sciences North, Sudbury, Ontario Canada; 2 Faculty of Education and Health, School of Kinesiology and Health Sciences, Laurentian University, Sudbury, Ontario Canada; 3 Northern Ontario School of Medicine, Sudbury, Ontario, Canada; 4 Northeastern Ontario Stroke Network, Sudbury, Ontario Canada

**Keywords:** Stroke, Mirror therapy, Education

## Abstract

**Background:**

Many therapeutic interventions are performed by physiotherapists to improve upper extremity function and/or activities of daily living (ADL) in stroke patients. Mirror therapy (MT) is a simple technique that can be self-administered by the patients with intact cognition following patient education by a skilled physiotherapist. However, the effectiveness of self-administered MT in post-stroke patients in upper extremity function remains unclear. Therefore, the objective of this study is to examine the effectiveness of MT in improving upper extremity function and recovery in acute stroke patients.

**Methods:**

This study is a single-center, prospective, randomized, open-label, controlled trial with blinded outcome evaluation (PROBE design), in which a total of 36 eligible acute stroke patients will be randomly assigned to control (n=18) and experimental group (n=18). Participants in the control group will receive regular rehabilitation interventions whereas participants in the experimental group will receive MT education in addition to their regular interventions for 4 weeks.

**Study outcome:**

The primary outcome measure will be upper extremity function that will be measured using the Fugl-Meyer Assessment scale and the Wolf Motor Function Test. The secondary outcome measure will be behaviors related to ADL as estimated using the Modified Barthel Index. Outcome measures will be assessed at baseline and at 4 weeks post-rehabilitation intervention/MT.

**Results:**

A two-way repeated analysis of variance (ANOVA) with time and group effects will be used to analyze between-group differences. The level of significance will be set at *P* < 0.05.

**Conclusion:**

The results of the study will provide critical information to include self-administered MT as an adjuvant to regular interventions and may facilitate recovery of the upper extremity function of stroke patients.

**Trial registration:**

ClinicalTrials.gov NCT04542772. Registered on 9 September 2020. Protocol version: Final 1.0.

## Background

Stroke is a sudden loss of brain function caused by focal cerebral ischemia or hemorrhage resulting in neurological impairments [1]. According to available statistics from 2012 to 2013, the number of stroke survivors in Canada is equivalent to the population of New Brunswick. It is the third leading cause of death and physical disability in Canadian adults [2]. Unfortunately, up to 78% of stroke survivors have impaired upper extremity function resulting in diminished activities of daily living [3]. Evidence shows that stroke survivors with initial upper extremity weakness can regain function using therapeutic interventions [4]. The Canadian Stroke Best Practice Guidelines (CSBPG) outlined specific therapies, such as range-of-motion exercises, mental imagery, functional electrical stimulation, mirror therapy (MT), constraint-induced movement therapy, virtual reality, strength training, and bilateral arm training, to improve upper extremity function [5]. While the majority of these therapeutic procedures are administered by skilled professionals, mirror therapy is a simple and inexpensive technique that can be self-administered by the patients with intact cognition.

In MT, the patient is seated with a lap tray and a mirror is placed in between the arms in midsagittal plane with the mirror facing the unaffected arm. The patient is asked to move the unaffected arm while looking at the mirror. The patient perceives the reflection in the mirror as his paralyzed arm and the brain receives a stimulus as if the patient is moving his affected arm. This visual stimulus supports motor rehabilitation by capitalizing on the strong connection between visual input and the premotor cortex [5, 6]. The effectiveness of MT in upper extremity function has been well explored in the past [7–11].

## Critical review

In an effort to identify gaps in the literature and to identify the parameters that can be used for our study, published systematic reviews and meta-analyses on the use of mirror therapy on upper extremity function in post-stroke patients were reviewed. A total of 49 studies were identified in six systematic reviews [12–17]. We categorized those 49 studies as acute/early sub-acute stroke (< 4 weeks), sub-acute stroke (< 3 months), and chronic stroke (> 3 months) based on the initiation of intervention from stroke onset [7]. Of these 49 studies, five studies (Table 1) were conducted on acute/early sub-acute stroke patients and were included for critical analysis (Table 2) [8–11, 18]. We found that the included five studies had limited sample sizes, weak methodology, and MT intervention were therapist guided. The frequency and duration of the MT intervention ranged from 5 days/week, 30 min/session for 2 weeks to 5 days/week, and 30 min/session for 6 weeks. The type of exercise training varied across all 5 trials. Bilateral arm training was included in one study [7], unilateral movement in two studies [8, 11], MT combined with constraint-induced movement therapy in one study [10], and MT combined with neuro-muscular electrical stimulation in one study [9]. In all five studies, it was concluded that MT was effective in improving the upper extremity function of stroke patients.

**Table 1:**
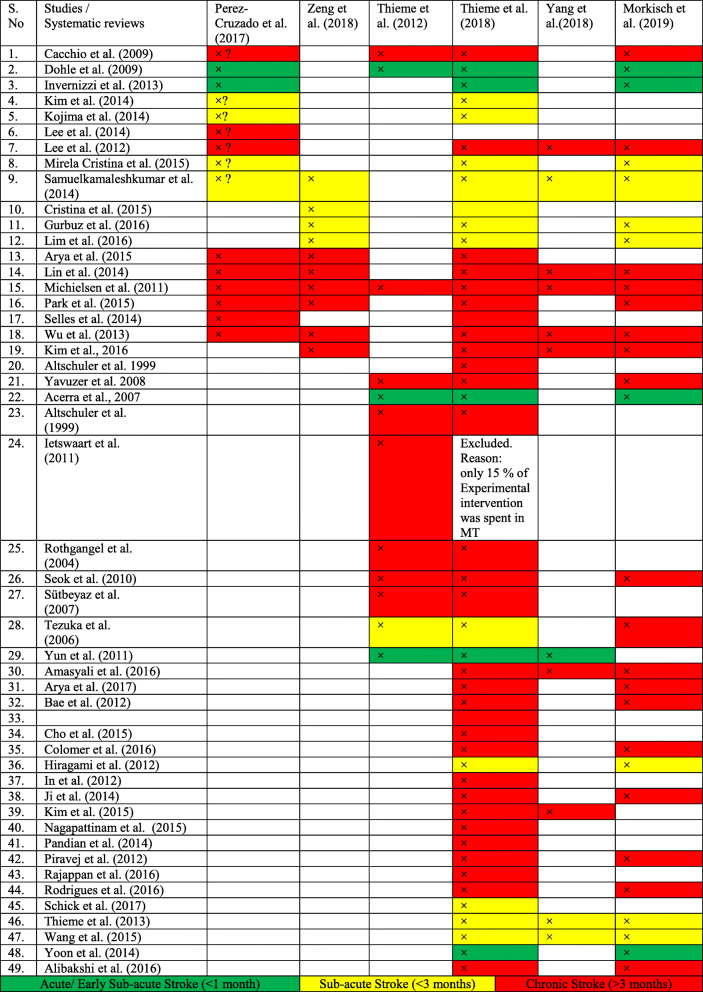
Primary studies included in systematic reviews

**Table 2:** Sample size, risk of bias, and PEDro scores

Reference	Sample size and dropouts	Cochrane risk of bias tool				PEDro score	Who led MT intervention?
		Random allocation (selection bias)	Allocation concealment (selection bias)	Incomplete outcome data (attrition bias)	Blinding of outcome assessment (detection bias)		Therapist/ Patient
Dohle et al., 2009 [8]	48 and 12	Low	Low	High	Low	7/11	Therapist
Invernizzi et al., 2013 [9]	26 and 1	Low	Low	High	Low	6/11	Therapist
Yun et al., 2011 [10]	60 and not reported	Low	High	High	High	5/11	Therapist
Yoon et al., 2014 [11]	26 and not reported	Low	High	High	High	5/11	Therapist
Cristina et al., 2015 [16]	15 and not reported	Low	High	High	Low	5/11	Therapist

Reported: low risk, not reported: high risk

The effectiveness of MT was summarized in one of the recent Cochrane reviews which included randomized controlled trials (RCTs) and randomized cross-over trials comparing MT and other interventions. The results suggested moderate-quality evidence that MT has a significant effect on motor function with SMD 0.47, 95% CI 0.27–0.67 for all outcome measures and mean difference of 4.32, 95% CI 2.46–6.19 for Fugl-Meyer Assessment upper extremity [19]. The authors suggested that MT should be considered, at least, as an adjuvant to conventional rehabilitation to improve upper extremity motor impairments and ADL of stroke survivors [17].

Morkisch et al. performed a secondary meta-analysis on a Cochrane review for MT to detect which parameters of the therapeutic protocols influence the effect of MT in upper extremity rehabilitation in post-stroke individuals. They found that unilateral arm movement execution without manipulation of objects with a larger mirror was found to be more effective in improving motor function than bilateral movement including manipulation of objects with using a smaller mirror [12]. The use of a larger mirror (50 x 40 cm) lessens the distraction and increases unilateral visual feedback. In addition, the larger mirror allows incorporation of shoulder movements and shaping (adaptation of the content of the therapy) [18]. Evidence shows that mirrored movements of the unaffected limb resulted in sufficient neural activation, without movement of the paretic limb [20, 21].

Patient self-managed education program has been shown to be effective in improving patient outcomes and in reducing treatment costs in other clinical conditions [22]. In addition, there is evidence that self-delivered MT intervention is feasible and improves patient outcomes in other clinical conditions [23]. Although the effect of patient education and effectiveness of MT are extensively explored in separate studies, the effectiveness of self-administered MT has not been explored in acute stroke patients. Also, literature shows that rehabilitation interventions are crucial during the first 4 weeks post-stroke as it is a critical time for neural plasticity and recovery [7]. Therefore, the objective of the present study is to examine the effectiveness of MT in improving upper extremity function and recovery in acute care settings.

## Material and methods

### Study design

A single-center, prospective, randomized, open-label, controlled trial with blinded outcome evaluation (PROBE design) will be used to evaluate the effectiveness of self-administered MT on upper extremity functions of acute/or early sub-acute stroke patients.

### Study setting

The study will be conducted at the acute stroke and intensive rehabilitation units at one of the Ontario hospitals between July 2021 and June 2022. We assessed feasibility of single-center study by reviewing The Discharge Abstract Database (DAD) which revealed approximately 300 stroke annual stroke admissions for the last two fiscal years. This will allow sufficient patient recruitment (calculated below) over 1 year period for the study.

### Study participants

A stroke team member at the hospital will identify potential patients, and a physiotherapist will assess patient eligibility based on the following inclusion: (1) > 18 years of age; (2) any gender; (3) admitted with a diagnosis of stroke with onset within 2 weeks; (4) Brunnstrom stages of stroke recovery between 2 and 4 for the upper extremity; (5) able to follow directions; (6) no severe cognitive impairments that could interfere with patient participation; (7) consent to treatment by the patient; and (8) Alpha-Functional Independence Measure (Alpha-FIM) score of > 40. Patients will be excluded based on several criteria: (1) medically unstable patients; (2) lack of motivation; (3) recurrent/chronic stroke; (4) recent upper extremity musculoskeletal injuries with movement restrictions; (5) receptive or global aphasia; (6) delirium; (7) unilateral neglect; and (8) visual field defect.

### Group assignment

#### Sample size estimates

The sample size and effect size were calculated based on a previously published study [24], using the G* power software. A sample of 18 will be required in each group to detect a difference in the primary outcome measure (FMA-UE) with an effect size (Cohen’s d) of 1.15 and a statistical power of 0.80 at a one-sided type one error of 0.05.

**Fig. 1:**
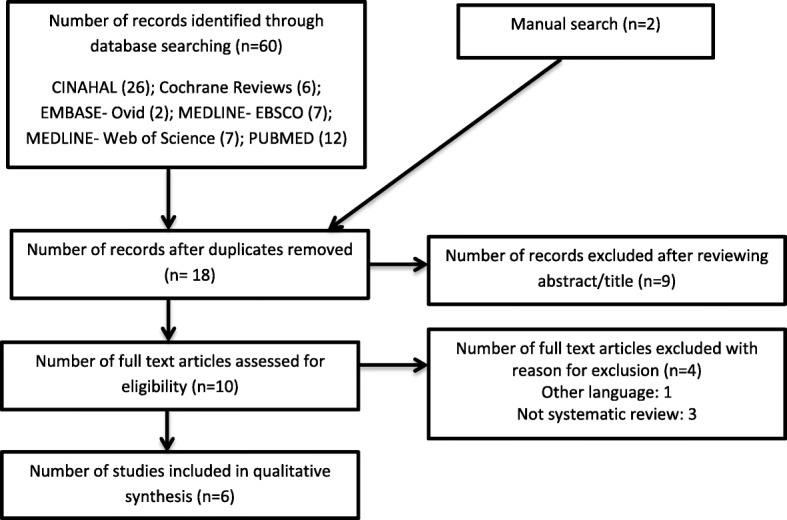
Preferred Reporting Items for Systematic Reviews and Meta-Analysis (PRISMA) used to identify studies

Based on the above calculation, a total of 36 patients will be recruited and assigned to the MT (n = 18) or control group (n =18) using a random sampling method. Randomization will be done prior to the baseline assessment with a statistical software program that generates this sequence. Random numbers will be assigned according to the order of patients’ admission to the hospital. To *conceal allocation*, random group allotment will be managed by a social worker who will not be involved in the assessment or intervention to minimize the confounding and contamination. Refer to Fig. 1 for the study process.

### Blinding

Pre- and post-test assessments will be performed by a physiotherapist who will be blinded to group allocation (assessor blinding). Patient education will be provided by a research assistant who will neither be involved in outcome assessment nor with implementing regular rehabilitation interventions. The research assistant will ensure that the patient understands the parameters of the exercise intervention, mirror placement, and unilateral arm execution (nonparalytic arm should be moved and not the paralyzed arm) and masters the MT technique.

### Interventions

Both the experimental and the control groups will receive the standard-of-care, multidisciplinary rehabilitation intervention based on their needs and tolerance, whereas patients in the experimental group will self-administer MT for an additional 30 min. During MT education, the patients will be educated or shown how to perform MT and the patients will complete the recommended exercises on their own for 5 days a week for 4 weeks. The research assistant will aid with setup if needed. As recommended in the standard MT procedures, patients will be asked to remove all jewelry and to sit on the chair or wheelchair in front of the table. A mirror will be placed on the table at a perpendicular angle, and its reflecting surface will face the non-paretic limb. The paretic limb will be positioned behind the mirror, and the non-paretic limb will be placed in front of the reflecting surface of the mirror. Patients will be asked to look at the mirror while doing exercises with the non-paretic limb. This activity will provide visual feedback to the brain like the patient moving the paretic limb. The patients in the MT group will be educated to perform the following several movements on the non-paretic side while looking at the mirror: (1) metacarpophalangeal (MCP) flexion, extension, and abduction; (2) fist and release; (3) wrist ulnar and radial deviations; (4) wrist flexion and extension; (5) forearm pronation and supination; (6) elbow flexion and extension; (7) shoulder flexion; and (8) shoulder abduction. Each movement will be performed for 10 repetitions and 2–3 sets (depending on patient tolerance) for a total of 30 min/session for 5 days a week for a period of 4 weeks. Patients in the experimental group will be given an exercise logbook to record exercise compliance. The logbook will include date, duration of MT, types of exercises (e.g., shoulder movements, elbow movement, wrist movement, and finger movements). Patients will be asked to record the above every single time they do the exercises. Exercise compliance will be considered as adequate if patients complete 80% of MT protocol (e.g., 25 min per day for 4 days a week for 3 weeks).

Participant bias will be minimized by assuring the participant that the quality of rehabilitation will not be affected by group assignment and by explaining that the trial aims to analyze whether the addition of MT education will have an additional beneficial impact on functional outcomes. The research will be initiated in an acute stroke unit and will be carried over in an intensive rehabilitation unit of the same hospital. We are not anticipating any confounding variables at this time during the patient transfer process as this happens within the same hospital and the patients in the experimental group will be self-administering MT only after the regular rehabilitation hours without interrupting any of the exercises or education that are regularly provided by therapists at intensive rehabilitation unit. The mirror for MT will be provided to the patient during the initial assessment (pre-test assessment) that the patient will take it with them to the rehabilitation unit and will be returned at the end of 4 weeks post-assessment.

### Outcome measures and data collection

The primary outcome measures of interest are the level of upper limb motor impairment and functional performance. These variables will be measured using the Fugl-Meyer Assessment-upper extremity (FMA-UE) and the Wolf Motor Function Test (WMFT), respectively. Both FMA-UE and WMFT are reliable and valid outcome measures and are used consistently in MT research [25]. Moreover, the Stroke Edge Task Force of the Academy of Neurological Physical Therapy (American Physical Therapy Association) recommends FMA-UE and WMFT for patients who have had a stroke and who are undergoing acute care [26]. The secondary outcome measure will be ADL measured by the Modified Barthel Index (MBI), a reliable and valid tool for assessing ADL in stroke survivors and used in MT research.

**Fig. 2:**
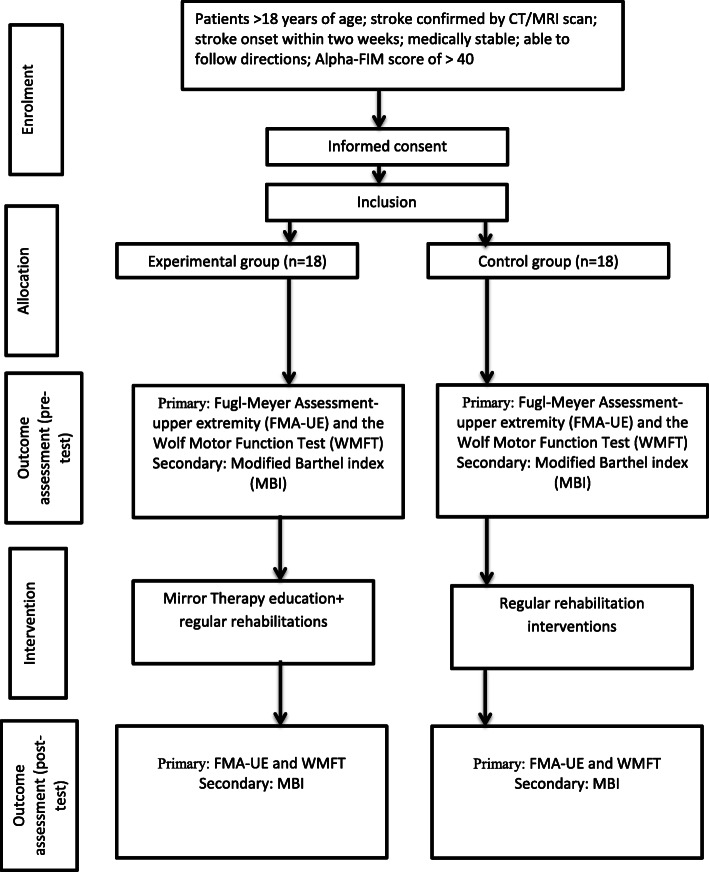
Flow chart representing study process

A physiotherapist will assess the above outcome measures twice: (1) pre-test assessment at the beginning of the trial and (2) post-test assessment at the end of the trial. Before the study, the assessor will be trained in assessing all outcome measures and will be tested with two patients to assure standardization. The assessors will be trained by one of the investigators of this study (VR) who is a physiotherapist specialized in stroke rehabilitation and experienced in administering the above outcome measures. In addition, demographic characteristics, such as age, gender, handedness, Alpha-FIM score, Brunnstrom stages of motor recovery, type of lesion, side of hemiplegia, and the onset of stroke, will be collected during the initial assessments (Fig. 2).

### Participant timeline

Participant timeline was developed according to the SPIRIT guideline and is presented in Table 3.

**Table 3:** Timeline of screening, recruitment, randomization, interventions, and assessments

Time point	Study period		
	0 week (w)	4 w	Closeout (4 w)
Enrollment			
Eligibility screen	x		
Informed consent	x		
Allocation	x		
Intervention			
Experimental group	x	x	
Control group	x	x	
Assessments			
Demographics	x		
FMA-UE	x	x	
WMFT	x	x	
MBI	x	x	

*FAM-UE* Fugl-Meyer Assessment-upper extremity, *WMFT* Wolf Motor Function Test, *MBI* Modified Barthel Index

Rehabilitation interventions are crucial during the first 4 weeks post-stroke as it is a critical time for neural plasticity and recovery [17]. Therefore, the present study will be initiated within 48 to 72 h of patient admission in the acute care unit and will be continued for 4 weeks following the initial assessment. Following admission to an acute stroke unit, screening and informed consent and baseline outcome assessments will be completed within 48 to 72 h. The mirror therapy education will commence immediately after the baseline assessment and end in 4 weeks.

### Statistical analysis

#### Data analysis

Data will be analyzed using the SPSS software version 23. Demographic characteristics and baseline scores will be compared using the independent *t*-test and the chi-squared test for continuous variables (such as age and time since stroke) and dichotomous variables (gender, dominance, affected side, and type of lesion), respectively. A two-way repeated analysis of variance (ANOVA) with time and group effects will be used to analyze between-group differences. We will administer both intention-to-treat and per-protocol analyses to determine the effectiveness of the proposed intervention [27]. The level of significance will be set at *P* < 0.05. Patients will be considered as being compliant if they perform at least 80% of the original MT protocol. The compliance will be reported using the four ways recommended by Hawley-Hague et al. (i.e., completion, attendance, duration adherence, and intensity adherence) [28]. In addition, CONSORT will be used to report this RCT to facilitate reading and quality assessment.

### Limitations

A limitation of this study is having two primary outcome measures which affects the power of the study. However, we decided to include both the outcome measures as we are interested in exploring the effectiveness of MT in improving motor impairment and motor function.

## Safety and adverse event monitoring

A data safety monitoring board will be notified outlining adverse events that occur from this study, from baseline assessment through to completion of the final evaluation.

## Stopping rules and early termination

Participants will be removed from the study if they want to withdraw themselves from the study. Participants’ data will be removed and will not be included for analysis if they request the withdrawal of their data before entering it for data analysis.

## Discussion/conclusion

This will be the first study exploring the benefits of patient education on MT in acute stroke patients. We expect that the results of the study will provide critical information to plan MT as an adjuvant to regular interventions for acute stroke patients. This inexpensive treatment technique may improve patient participation and therapy time which may promote robust recovery of the upper extremity function of stroke patients.

## Trial status

The current protocol version is “Final 1.0.”, as of September 9, 2020. A total of 36 patients will be recruited for the study. As of May 19, no patients were recruited for the study. Recruitment is estimated to begin in July 2021 and end in June 2022.

## Data Availability

Additional data and documents are available on request.
